# Influence of Silica-Aerogel on Mechanical Characteristics of Polyurethane-Based Composites: Thermal Conductivity and Strength

**DOI:** 10.3390/ma14071790

**Published:** 2021-04-05

**Authors:** Jeong-Hyeon Kim, Jae-Hyeok Ahn, Jeong-Dae Kim, Dong-Ha Lee, Seul-Kee Kim, Jae-Myung Lee

**Affiliations:** 1 Hydrogen Ship Technology Center, Pusan National University, Busan 36241, Korea; honeybee@pusan.ac.kr (J.-H.K.); kfreek@pusan.ac.kr (S.-K.K.); 2 Hyundai Heavy Industries Co., Ltd., Ulsan 44032, Korea; hllo0130@naver.com; 3 Department of Naval Architecture and Ocean Engineering, Pusan National University, Busan 46241, Korea; jeongdae3416@pusan.ac.kr (J.-D.K.); dongha2@pusan.ac.kr (D.-H.L.)

**Keywords:** polyurethane foam, cryogenics, mechanical properties, thermal conductivity, silica aerogel

## Abstract

Polyurethane foam (PUF) has generally been used in liquefied natural gas (LNG) carrier cargo containment systems (CCSs) owing to its excellent mechanical and thermal properties over a wide range of temperatures. An LNG CCS must be designed to withstand extreme environmental conditions. However, as the insulation material for LNGC CCSs, PUF has two major limitations: its strength and thermal conductivity. In the present study, PUFs were synthesized with various weight percentages of porous silica aerogel to reinforce the characteristics of PUF used in LNG carrier insulation systems. To evaluate the mechanical strength of the PUF-silica aerogel composites considering LNG loading/unloading environmental conditions, compressive tests were conducted at room temperature (20 °C) and a cryogenic temperature (−163 °C). In addition, the thermal insulation performance and cellular structure were identified to analyze the effects of silica aerogels on cell morphology. The cell morphology of PUF-silica aerogel composites was relatively homogeneous, and the cell shape remained closed at 1 wt.% in comparison to the other concentrations. As a result, the mechanical and thermal properties were significantly improved by the addition of 1 wt.% silica aerogel to the PUF. The mechanical properties were reduced by increasing the silica aerogel content to 3 wt.% and 5 wt.%, mainly because of the pores generated on the surface of the composites.

## 1. Introduction

The consumption and demand for natural gas (NG), an environmentally friendly fuel, are increasing owing to environmental regulations in the shipbuilding industry. According to International Maritime Organization (IMO) environmental regulations, which are emerging as a major factor in the international shipbuilding and shipping industry, the permitted level of sulfur oxide emitted from ships passing through all the sea areas of the world will be reduced from 3.5% to 0.5%. An additional IMO strategy for reducing greenhouse gas emissions was implemented in April 2018, which required at least 50% of gases emitted by ships to be accurately identified by 2050 [1]. Therefore, shipowners must replace conventional fuels with liquefied natural gas (LNG) to abide by the imposed regulations. This trend has led to an increase in the demand for liquefied natural gas carriers (LNGCs), which can efficiently transport NG in liquid form at a cryogenic temperature of −163 °C and pressure of 1.1. The LNGC consists of cargo containment systems (CCSs), which are the core technology in LNGCs for the safe transport of LNG. The CCSs must withstand various loads and cryogenic conditions because the insulation system of the CCS is in direct contact with the LNG and is thus continuously exposed to the cryogenic environment during LNG transport. Therefore, the material used in CCSs should be usable in cryogenic temperature conditions.

Polyurethane foam (PUF) has been generally used in LNGC CCSs owing to its excellent mechanical characteristics and thermal properties over a wide range of temperatures; even when the temperature gradient is varied, the volume of the material does not change significantly, and the size corresponding to the stable state is maintained at cryogenic temperatures with low production costs [2]. Thus, PUF is extensively used in various fields, particularly in LNG tanks, for insulation purposes [3]. In recent decades, significant research has been conducted on the mechanical characteristics of PUF applied to LNG insulation systems. Han et al. carried out a repetitive impact test of glass-fiber-reinforced polyurethane foam (RPUF) and PUF to evaluate their failure criteria and structural applicability using dry-drop equipment. The RPUF showed improved resistance to permanent deformation and crack propagation [4]. Lee et al. proposed a material constitutive equation by analyzing the mechanical properties of PUF under compressive load and demonstrated the effect of the apparent density in a compression test [5]. Park et al. carried out compressive tests of RPUFs and compared them in terms of the blowing agent (CO_2_ and HFC-245fa), temperature, and strain rate. On comparison of the compressive strengths of RPUF with the two blowing agents at two temperature conditions (20 °C and −163 °C), the RPUF using the HFC-245fa blowing agent showed a higher compressive strength at room temperature, and the opposite result at the cryogenic temperature [6].

**Figure 1:**
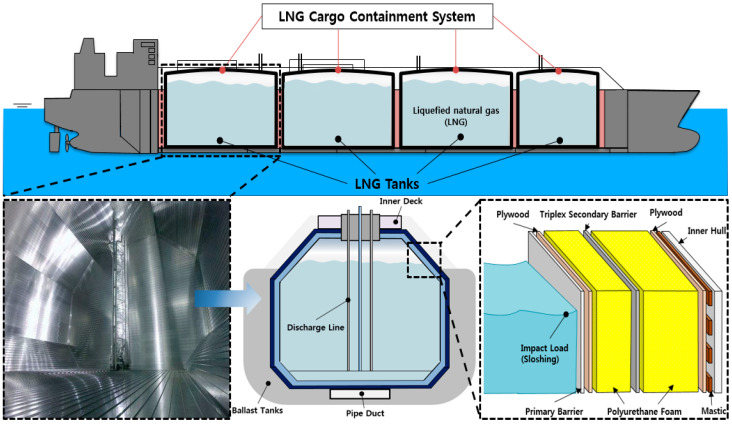
Schematic of liquefied natural gas cargo containment system (LNG CCS) in LNG carrier and insulation system under various loads especially for sloshing owing to 6-degrees-of-freedom motions and environmental conditions.

However, as an insulation material for LNGC CCSs, PUF has two major limitations: its strength and thermal conductivity. Owing to the ocean wave conditions during the operation of the LNGC, the internal insulation system is subjected to fluid impact loads, such as sloshing of the LNG induced by the wave motion, as shown in Figure 1. The structural integrity of the insulation materials must be maintained under impact loading; therefore, RPUF, which has a higher strength and elastic modulus than neat PUF, is frequently used in LNGC CCSs to obtain sufficient mechanical strength to withstand the sloshing impact loads [7]. However, issues such as cracks and LNG fuel leaks in the internal insulation system remain despite the improvement in the compressive strength of RPUF by the glass fibers. Furthermore, the thickness of the RPUF must be increased to provide sufficient heat insulation in the system owing to the high thermal conductivity of the glass fibers. This reduces the storage space occupied by LNG, and the weight of the ship increases, resulting in a reduction in economic efficiency. To overcome these shortcomings, numerous studies have been carried out to improve the material properties of PUF by adding nanoparticles that can modify the cellular structure and improve the properties of the solid matrix through interactions.

Cai et al. studied the mechanical and thermal properties of graphene oxide (GO) mixed with polyurethane. The addition of GO during synthesis led to a substantial enhancement in both the stiffness and toughness of the PUF. The modulus of elasticity was improved by approximately seven times on the addition of 4 wt.% GO [8].

Silica aerogel, a nanoporous, lightweight material, is known for its super-low thermal conductivity in thermal insulation applications [9]. In particular, silica aerogels have been widely studied for their applicability as materials for the advancement of the energy, environmental material, and electronic industries because of their unique thermal, electrical, and optical properties. The use of silica aerogel as a catalyst has been extensively discussed owing to its low density, high specific surface area, and porous structure characteristics [10]. Further, silica aerogel is used to reduce thermal losses in wide-ranging applications such as insulation paints and boards in the construction and aerospace industries because of its excellent thermal insulation performance, high porosity, and low density compared with its volume [11,12]. For these reasons, we adopted silica aerogel powder for the synthesis of PUF to improve the heat insulation performance.

Cimavilla-Roman et al. reported the influence of silica aerogel particles on the foaming process and cellular structure of rigid PUF. The results showed that the addition of low contents of aerogel reduced the conversion of isocyanate. However, the foam with high contents of aerogel did not change the reaction balance [13]. Bonab et al. synthesized silica aerogel/polyurethane hybrid nanocomposite foams. The results showed that with the increase in nanoparticle content, the cell size distribution was broadened, and cell density decreased. In addition, hydrophilic silica aerogel improved the homogeneity of cell mechanical properties of PUF nanocomposite foams [14]. Cho et al. reported flexible and coatable insulating silica aerogel/polyurethane composites. The results showed that the thermal insulation properties of aerogel–polyurethane composites were enhanced by a 72% reduction in thermal conductivity upon 30 wt.% aerogel loading. Furthermore, these results were theoretically verified by a micromechanics-based thermal conductivity model [15]. Although several studies aiming to enhance the performance of PUF have been carried out, most of them focused on compressive mechanical and thermal tests at room temperature (25 °C). Sufficient compressive test data at cryogenic temperatures (−163 °C) to evaluate the mechanical properties in LNGC environmental conditions do not exist. As mentioned, in addition to the mechanical strength, the insulating capacity should be considered.

In this study, five types of PUFs were prepared using silica aerogels as additives to investigate the effect of silica aerogels on the material characteristics of PUF. The compression tests of the PUF-silica aerogel composites were conducted at room temperature (20 °C) and cryogenic (−163 °C) temperatures to evaluate the compressive strength. To determine the insulation performance, the thermal conductivity was measured for various weight ratios of the silica aerogels in comparison with the PUF. In addition, through field emission scanning electron microscopy (FE-SEM) analyses, the cellular structures of the composites were observed to compare the material characteristics and the cells.

## 2. Experimental Preparations

### 2.1. Experimental Scenario

In this study, compression and thermal conductivity tests were conducted to evaluate the effects of the added silica aerogels on the mechanical and thermal properties of PUF. The compression test was performed until the thickness of the test specimen was reduced to 85% of the initial value at a strain velocity of 2.5 mm/min, corresponding to 10% of the initial thickness and per minute of the test specimen according to KS M ISO844 [16]. Two test temperatures were chosen based on the cryogenic temperature (−163 °C) during LNG loading conditions and at room temperature (20 °C) during offloading conditions in the LNGC CCSs. When the cryogenic compression test was conducted, the compression specimens were compressed by stainless steel compression jigs in the cryogenic compression chamber at a temperature of −163 °C. Therefore, before the tests were started, precooling was carried out for 1 h to obtain the thermal equilibrium of the specimens because the inside and outside temperatures of the specimen were different. In addition, thermal conductivity tests at room temperature (20 °C) using the heat flow meter (HFM) were conducted to investigate the effect of the added silica aerogels.

### 2.2. Raw Materials

The base material for the PUF composites consisted of three components: a polyether polyol (Billyol LI-110 system), methylene diphenyl diisocyanate (MDI, PAPI 27), and a blowing agent (HFC-245fa, CAS Number: 460-73-1), all obtained from Korea Polytech Ltd., Gimpo, Korea. As additives in the MDI, the silica aerogels had a white color, low weight, and super-low thermal conductivity. Table 1 lists the technical data of the silica aerogel [17].

### 2.3. Preparation of PUF-Silica Aerogel Composites

To investigate the effect of the silica aerogel on the PUF mechanical and thermal insulation performance, PUF and silica aerogel were fabricated using PUF raw materials (polyol, MDI, and blowing agent HFC-245fa) with silica aerogels. Table 2 shows the various ratios of the raw materials used for the PUF-silica aerogel composites. To investigate the effects of the silica aerogel on the mechanical performance of the PUF and to obtain the general characteristics of the silica aerogel PUF, 1, 3, and 5 wt.% silica aerogel were arbitrarily selected.

Because a constant ratio of the polyol, MDI, and blowing agent of 1000:1160:50 was used, the only variable parameter for the fabrication process of the composites was the weight percentage of the silica aerogels. The preparation process of the PUF-silica aerogel composites was as follows. First, for homogeneous dispersion of the silica aerogel in the MDI, it was thoroughly mixed by a homogenizer with the MDI because MDI had a lower viscosity than the polyol. Then, the polyol and blowing agent were added to the mixed suspension of silica aerogels and isocyanate. Considering the rapid gel time at which the mixed solution solidified, it was dispersed at a speed of approximately 4500 rpm for 60 s using the same homogenizer used to prepare the PUF-silica aerogel mixture solutions. Finally, the blended mixture was poured into an open-box-type mold and freely foamed for 24 h at room temperature (20 °C), reaching a thickness of 25 mm.

### 2.4. Experimental Apparatus

**Figure 2:**
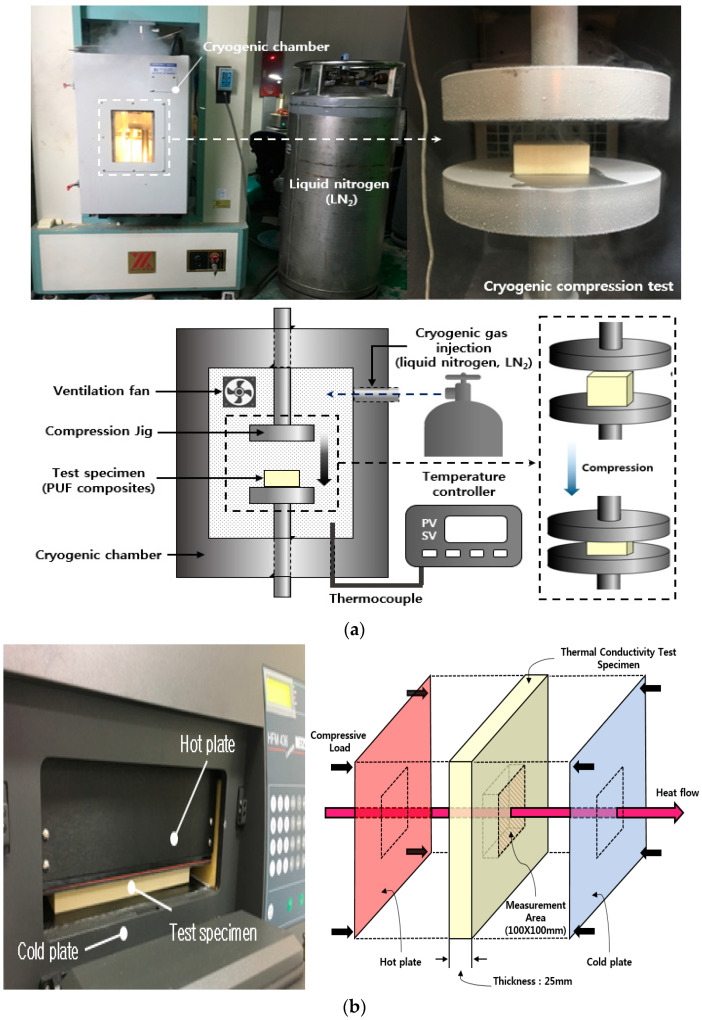
Experimental apparatus and schematic illustration: (**a**) universal testing machine for compression test at room (20 °C) and cryogenic temperature (−163 °C) and (**b**) heat flow meter for measurement of thermal conductivity at room temperature (20 °C).

In this study, a homogenizer (T50 digital ULTRA-TURRAX, IKA, North Carolina, USA) was used to disperse the silica aerogels and isocyanates homogeneously by mixing the polyol and the blowing agent. To analyze the PUF-silica aerogel composite morphology, the composite microstructure was observed using a scanning electron microscope (FE-SEM SUPRA25, Carl Zeiss AG, Oberkochen, Germany). To evaluate its mechanical strength in each environment, a universal testing machine (KSU-5M, Kyoungsung Testing Machine Co., Ltd., Ansan, Korea) was used to perform compression tests. A cryogenic chamber was installed in the universal testing machine to perform the cryogenic compression tests. To create a cryogenic environment in the chamber, liquefied nitrogen gas was continuously injected to keep the chamber temperature low. When the internal temperature of the chamber reached the set temperature of −163 °C for the cryogenic compression tests, the liquefied nitrogen gas injection was controlled by an automatic control system to maintain the internal temperature. Figure 2a shows the compression test at room temperature (20 °C) and cryogenic temperature (−163 °C).

In this study, the thermal conductivity of the PUF-silica aerogel composites was measured using a heat flow meter (HFM 436, NETZSCH, Selb, Germany) according to ASTM C518 [18]. Figure 2b shows the heat flow meter (HFM) equipment and the measurement method for the thermal conductivity. The dimensions of the test specimen to obtain reliable data were as follows: width, length, and thickness of 280 mm, 280 mm, and 25 mm, respectively. The thermal conductivity test specimens were measured at room temperature (20 °C). The measurement method used was the flat-plate heat flow method. The thermal conductivity of the test specimen was measured by applying a temperature difference between the lower plate (10 °C) and the upper plate (30 °C) using a chiller as a cooling device while maintaining the temperature of the specimen at an average temperature of 20 °C. Additionally, a certain load was applied up and down so that the ambient air did not affect the thermal conductivity results.

## 3. Results and Discussion

### 3.1. Density of PUF-Silica Aerogel Composites

**Figure 3:**
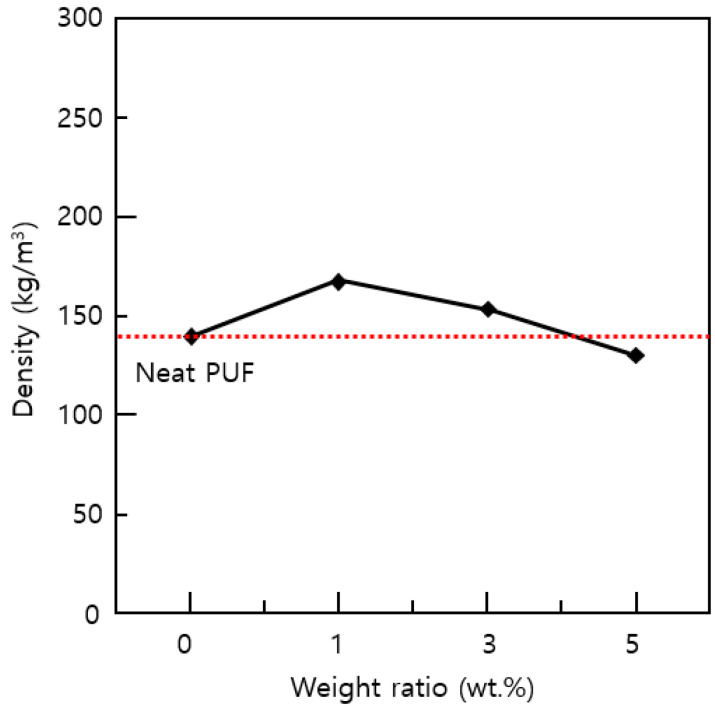
Comparison of average density for PUF-silica aerogel composites according to weight percent of silica aerogel nanoparticles.

Density is an important variable that greatly affects the thermal conductivity and in particular the mechanical properties of polymeric foams [19,20]. Therefore, when performing compression tests and thermal conductivity tests, the density should be characterized. The measurement results of the density of the PUF-silica aerogel composites are shown in Figure 3. For each weight percent of silica aerogels, the resulting values represent the average density for the four specimens. Before being processed into a specimen for compression and thermal tests, the bulk size of the PUF-silica aerogel composites had different heights in the same container frame used for preparation depending on whether silica aerogels were added.

**Figure 4:**
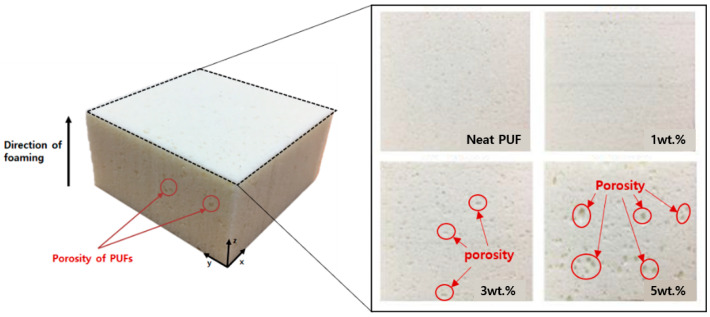
Photographs of test specimens for compression tests and top surface photographs for various weight ratios of silica aerogels.

Thus, for example, the total foaming height of the 1 wt.% silica aerogel before the cutting process was relatively low compared to the neat PUF of 0 wt.%. This means that a small quantity of silica aerogel affected the materials for PUF to agglomerate during the free forming time. This results in a higher density owing to the addition of silica aerogel, which acted as a nucleating agent for the PUF cell during the foaming process [21]. However, as the amount of silica aerogel increased, the density tended to decrease because pores were generated on the top surface, as shown in Figure 4. This is because in the case of nanoparticles, the dispersion decreases when the added amount is increased [5,7].

In addition, owing to the high porosity of the silica aerogel itself, more pores were generated when the added silica aerogel was increased from 3 wt.% to 5 wt.% owing to the addition of a relatively large amount of silica aerogel nanoparticles during the mixing process. When preparing compression and thermal conductivity test specimens, the pores replaced the space occupied by the PUF, resulting in a reduction in mass at the same volume and a lower density. Therefore, the densities of the PUF-silica aerogel composites were dependent on the silica aerogel content, which affected the mechanical properties and insulation performance. In the present study, the normalization equations for modulus of elasticity (*E_norm_*) and strength (*σ_norm_*) were used [22].

(1) $$\sigma_{norm}=\sigma_{exp}{\left(\frac{0.1}{\rho }\right)}^{2.1}\;E_{norm}=E_{exp}{\left(\frac{0.1}{\rho }\right)}^{1.7}$$

where *E_exp_* is the modulus of elasticity and *σ_exp_* is the strength determined from the stress-strain curve, and *ρ* is the density of the foam material.

### 3.2. Morphological Characteristics

The mechanical properties and thermal insulation performance of PUFs are highly dependent on the cell morphology. The properties of silica aerogels such as nanostructure, high porosity, and low density can affect the cellular structure of PUF. To analyze the cellular changes owing to the synthesis of PUF with the added silica aerogels, the cell morphologies of the PUF-silica aerogel composites were observed by field emission scanning electron microscopy (FE-SEM) at a magnitude of approximately 100 μm. The silica aerogels were not examined to be owing to the void diameter of 20 nm, as mentioned previously.

**Figure 5:**
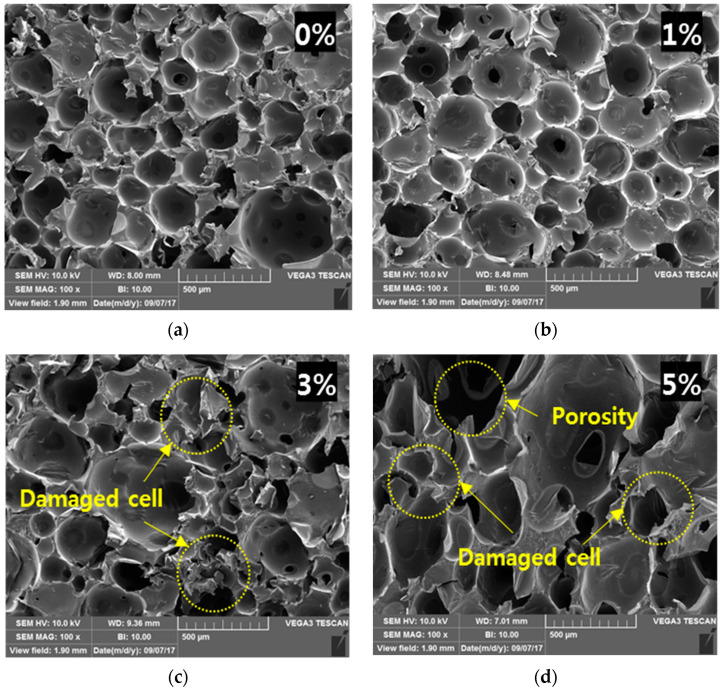
Cellular structure and morphologies with different silica aerogel concentrations in PUF-silica aerogel composites by FE-SEM micrograph: weight ratios of (**a**) 0 wt.%, (**b**) 1 wt.%, (**c**) 3 wt.%, and (**d**) 5 wt.%.

The surfaces were along the perpendicular direction of the foamed structures and are shown in Figure 5. The distribution of cells was more uniform, and the closed-cell morphology decreased with the addition of the silica aerogel. In general, neat PUFs of 0 wt.% have a closed-cell structure rather than an open-cell structure with poor insulation performance [16,23,24]. In addition, the cell allocation became typically homogeneous when nanoparticles such as nanoclay and graphene oxide content increased owing to the nucleation effect of the nanoparticles. However, the addition of more than the critical amount of the nanoparticles increased the destruction of the closed cells and worsened the dispersion between PUF and the nanoparticles [25,26]. Similarly, when the silica aerogel was added up to 1 wt%, the cell morphology and distribution had fewer nuclei compared with the neat PUF. As mentioned earlier, the reason for this is related to the nanoparticle nucleation effect, which can result in a uniform cell size.

However, by increasing the amount of silica aerogel to 3 wt.% and 5 wt%, it was found that the cell shape was irregular and often damaged, with the closed-type cells having a high insulation performance changing into collapsed cells that could not maintain their inherent heat insulating property. The cell formation process of the polymer foam was affected by the addition of nanoparticles according to previous studies [7]. Similar to previous studies, the dispersion relation of PUF and silica aerogel deteriorated with a variety of cell sizes, as shown in Figure 5c,d, and it was more difficult to form cells with uniform and closed morphology than the cells of the neat PUF when the content of the silica aerogel was greater than 1 wt%. In addition, it was confirmed that the porosity of PUF cells increased owing to the addition of silica aerogel. The results confirmed that the cell distribution and morphology were constant and stable when the weight ratio was relatively low.

### 3.3. Thermal Conductivity

For insulation materials such as PUF, evaluation of the thermal conductivity is a high priority. To prevent heat transfer in a heat-insulating material, the thermal conductivity should be low. Considering the stabilization time in which the blowing agent gas (HFC-245fa; thermal conductivity at 21.42 °C: 0.08894 W/m·K) generated during foaming was replaced with atmospheric air (thermal conductivity at 20 °C: 0.0253 W/m·K), the thermal conductivity was measured by HFM 12 days after manufacturing the bulk specimen to ensure accurate thermal conductivity measurements of the fabricated PUF-silica aerogel composites without the effect of the blowing agent [27]. Silica aerogel has superior heat insulation performance compared to PUF, so it was expected that the thermal conductivity would decrease during an increase in the content of silica aerogel. Contrary to expectations, the thermal conductivities of the PUF-silica aerogel showed a somewhat increasing trend with increasing wt.% of silica aerogel. Table 3 shows the thermal conductivity of PUF-silica aerogel composites at room temperature. When only 1 wt.% of silica aerogel was considered, the thermal conductivity was measured to be about 0.3% lower than that of neat PUF. However, when the densities of the specimens shown in Figure 3 were considered, the thermal conductivity was better in the case of 1 wt.% PUF-silica aerogel compared to the neat PUF considering the density of the PUF. In the case of the 3 and 5 wt.% PUF-silica aerogel, the thermal conductivity increased by 9.8% and 13%, respectively, compared to neat PUF at 20 °C. In general, the more uniform the shape and distribution of the cellular structure, the better the insulation performance. As a result of the microstructure being at 3 wt.% and 5 wt%, however, the number of pores increased, and cells were destroyed owing to nonhomogeneous dispersion during synthesis as well as free-forming time by the addition of the silica aerogel at 3 and 5 wt%. Thus, the insulation performance was improved only at a low level of silica aerogel rather than improving proportionally according to the added silica aerogel.

### 3.4. The Fourier Transform Infrared (FTIR) Analysis

**Figure 6:**
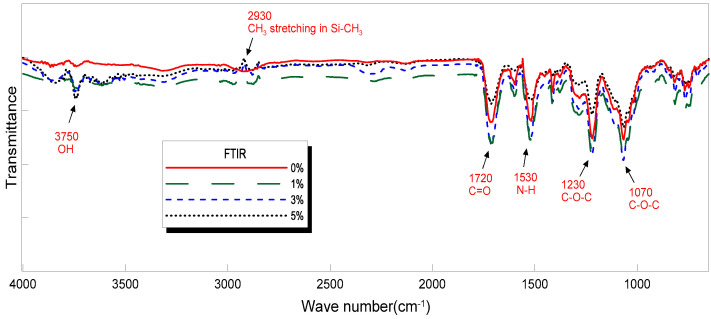
Peak analysis of FTIR spectra for PUF-silica aerogel composites.

In this study, structural changes, such as the material orientation and crystal shape of neat PUF and PUF-silica aerogel composites were confirmed by attenuated total reflectance Fourier transform infrared (FTIR) spectroscopy, which is a technique used to obtain an infrared spectrum of absorption or emission of a solid. The FTIR spectra of the PUF-silica aerogel composites are shown in Figure 6. Common peaks exhibited by the PUF were observed at 1720 cm^−1^, 1530 cm^−1^, 1230 cm^−1^, and 1070 cm^−1^. The PUF is formed by reacting a urethane bond (–OH, active hydroxyl group) with an isocyanate group (–N = C = O) in the isocyanate to generate heat, forming a PU with the structure of –NHCOO–. The FTIR analysis showed the formation of polyurethane bonds in all PUF samples. C–O stretching vibrations were observed at 1720 cm^−1^, N–H bending vibrations at 1530 cm^−1^, and asymmetric C–O–C stretching vibrations at 1230 cm^−1^. C–O–C antisymmetric stretching vibrations were observed at 1070 cm^−1^. It was confirmed that the area and height of peaks were commonly observed for an increase from 1 wt.% to 3 wt.% silica aerogel and then decreased for 5 wt.% silica aerogel. In addition, the peak length of the -OH stretching vibrations at 3750 cm^−1^ was longer than that at 0 wt%, and the presence of a peak point owing to the SiO_2_ component, which is the main component of the silica aerogels, was confirmed through asymmetric CH_3_ stretching in SiCH_3_ at 2930 cm^−1^.

### 3.5. Compression Tests

PUF is used because of its strength in various environments and conditions. The PUF used in LNG insulation systems requires a high compressive strength, as mentioned earlier. Therefore, it is important to evaluate the strength of the PUF by compression testing to determine the stress-strain relationship of the material, and tests were carried out on four samples under identical conditions to ensure the reliability and repeatability of the experimental data. The average value was used as a representative value.

**Figure 7:**
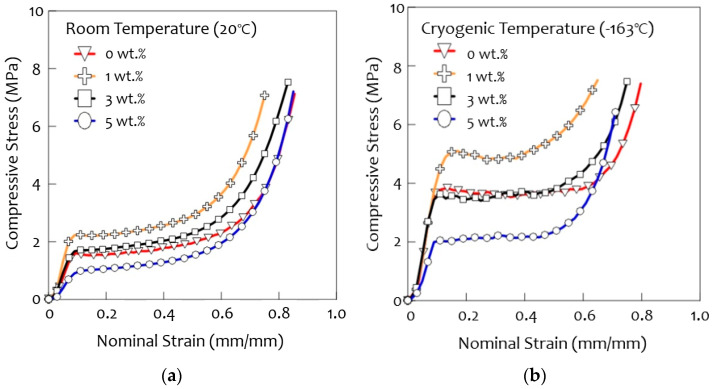
Stress-strain curve of compressive tests of PUF-silica aerogel composites at various weight ratios for (**a**) room temperature (20 °C) and (**b**) cryogenic temperature (−163 °C).

**Figure 8:**
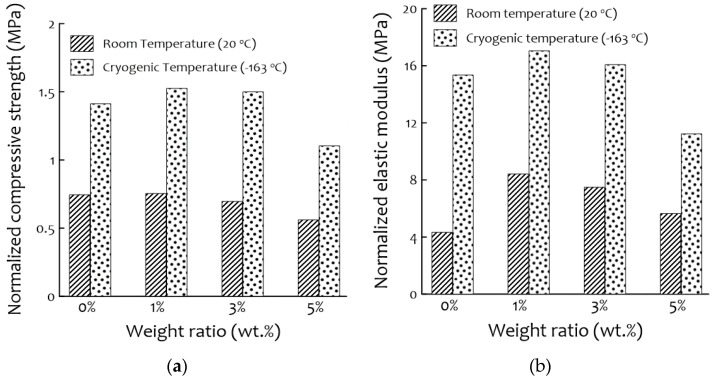
Temperature- and weight-ratio-dependent mechanical characteristics of PUF- silica aerogel composites: (**a**) normalized compressive strengths and (**b**) normalized elastic modulus.

The results of the compressive stress tests at room and cryogenic temperatures are shown in Figure 7. The compressive strength was higher at cryogenic temperatures (−163 °C) than at room temperatures (20 °C) for all samples, as was found in previous studies [28]. The highest compressive strength was observed for the 1 wt.% PUF-silica aerogel composite and the 3 wt.% sample, which show a curve similar to that of neat PUF (0 wt.%). In the case of the 5 wt.% of PUF-silica aerogel, the composite showed a sudden increase in compressive strength with the increase in nominal strain. This is because densification occurred early due to the large cell size deviation because the uniform cells support compressive loads better than ununiformed cells. Figure 8 shows the normalized compressive strength and elastic modulus. As shown in this figure, the normalized compressive strength of the 1 wt.% composite was the highest at all temperatures, and the normalized elastic modulus tended to exhibit the same trend as the compressive strength. Comparing the neat PUF at 0 wt.% and 1 wt.% of PUF-silica aerogel composite, the normalized compressive strength of the 1 wt.% composite increased by 1.1% at room temperature (20 °C) and 8% at the cryogenic temperature (−163 °C). However, the normalized compressive strength decreased at 3 wt.% and 5 wt.% compared with 1 wt.% of the PUF-silica aerogel composite because the amounts of the additives had a great influence on the mechanical properties. In particular, when a large amount of silica aerogel was added, a uniform cell morphology was not formed, which was suggested to be owing to the excessive addition deteriorating the dispersion in the PUF solutions, as mentioned earlier [20,29,30,31]. In addition, the pores generated by the deterioration of the dispersion relationship decreased the mechanical strength.

**Figure 9:**
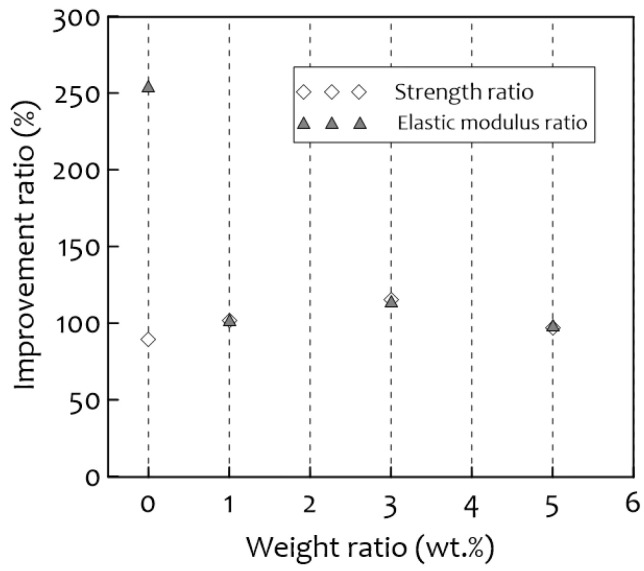
Compression of compressive strength and elastic modulus improvement ratios for temperature difference between cryogenic (−163 °C) and room (20 °C) temperatures.

Figure 9 shows the improvement ratio of the normalized compressive strength to the normalized elastic modulus for the temperature difference between room temperature and cryogenic temperature. In the case of the increment value, the weight percent of silica aerogels shows how much the mechanical strength was improved when the temperature was changed. The ratio between the mechanical compression strength at cryogenic temperature and room temperature was increased by considering the effect of low temperature to a similar level. The ratio of the elastic modulus for PUF-silica aerogel composites also tended to increase at a similar rate, and there was no significant difference, except for the 0 wt.% (neat) PUF. Figure 9 shows the test results of the compression strength and elastic modulus based on the ratio of the temperature difference. The compression strengths of the PUFs with 0, 1, 3, and 5 wt.% silica aerogel at cryogenic temperatures increased by approximately 89%, 102%, 115%, and 97%, respectively, compared to those at room temperature. The elastic modulus of the 0 wt.% (neat) PUF at cryogenic temperature increased by approximately 254% compared to that at room temperature, and those of the 1, 3, and 5 wt.% PUFs at cryogenic temperature increased by approximately 102, 115, and 99%, respectively. The normalized compressive strength and normalized elastic modulus of PUF-silica aerogel composites at 1 wt.% were much higher than those of neat PUF, but the latter reacted more sensitively to a variable temperature.

### 3.6. Fracture Characterization

**Figure 10:**
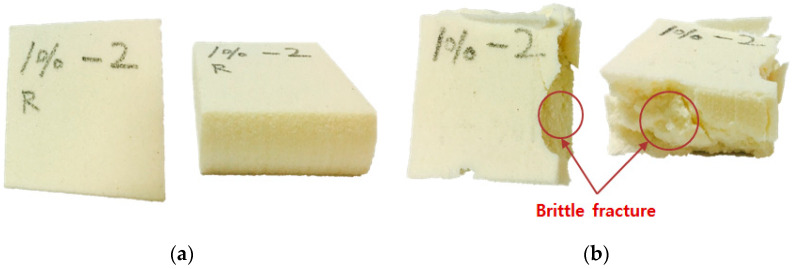
Fracture characteristics of PUF–silica aerogel (1 wt.%) composites owing to compression tests at (**a**) room (20 °C) temperature and (**b**) cryogenic temperature (−163 °C).

Photographs of the specimens after compression testing at 20 °C and −163 °C are shown in Figure 10. The temperature affected the mechanical properties such as the hardness and elasticity of the PUF-silica aerogel composites, so the fracture characteristics of the PUF depend on the temperature conditions. As a result, all specimens that were subjected to a cryogenic compression test exhibited a side-breaking phenomenon because of brittle fracture at low temperatures. The lower the temperature during the compression test, the more easily the PUF broke down, even for small forces [5]. However, at room temperature, no cracking occurred during the compression test, and the sample fully recovered its height.

## 4. Conclusions

PUF-silica aerogel composites were investigated to determine the effect of silica aerogels on the mechanical properties of PUF at room temperature and cryogenic temperature. The thermal conductivities and cell microstructures were also evaluated to identify the heat insulation characteristics according to the ratios of silica aerogel. The main results are summarized as follows.

∙ The optimal content of silica aerogel in this research was 1 wt.%, which led to a significant increase in compressive strength both at room temperature (20 °C) and cryogenic temperature (−163 °C).
∙ The cell morphology of PUF-silica aerogel composites was relatively homogeneous, and the cell shape remained closed at 1 wt.% in comparison with the other concentrations.
∙ The specific thermal conductivity of the 1 wt.% PUF-silica aerogel composites was lower because of the uniform and homogenous cell shape modification of the cellular structure and the extremely low thermal conductivity of the silica aerogel material, despite the increasing density, which had a significant effect on the insulation performance.
∙ The mechanical properties were reduced by increasing the silica aerogel content to 3 wt.% and 5 wt.%, mainly because of the pores generated on the surface of the composites. Because of the generated pores and nonhomogeneous cell distribution with silica aerogel dispersion in the PUF, the thermal insulation properties also decreased.

In summary, the mechanical and thermal properties were improved by the addition of 1 wt.% silica aerogel to the PUF. Consequently, considering the applicability of the material developed in this study to LNG insulation systems used in cryogenic environments, it has considerably high compressive strength and better heat insulation compared with neat PUF. The silica aerogel PUF showed brittle fracture characteristics at cryogenic temperatures, even though compressive strength and thermal conductivity showed better performance. In a future study, we will improve the mechanical performance and perform a variety of additional tests to verify the silica aerogel PUF’s specific characteristics.

## Figures and Tables

**Table 1:** Physical properties of silica aerogel nanoparticles.

Silica Aerogel	Contents
Particle size range	10–200 μm
Void diameter	20 nm
Pore volume	2.2–2.5 cm^3^/g
Particle density	70–150 kg/m^3^
Thermal conductivity	0.0018–0.02W/m·K at 25 °C
Surface area	300–350 m^2^/g
Porosity	90–99%

**Table 2:** Compositions of polyurethane foam (PUF)-silica aerogel composites.

Materials		Content	
		Weight (g)	Ratio (%)
Polyol Mixture		1000	-
Polymeric MDI		1160	-
HFC-245fa		50	-
Silica Aerogels	0 wt.% Neat PUF	0	0
	1 wt.% PUF-silica aerogel composites	22.1	1
	3 wt.% PUF-silica aerogel composites	66.3	3
	5 wt.% PUF-silica aerogel composites	110.5	5

**Table 3:** Evaluation of thermal conductivity of PUF-silica aerogel composites at room temperature (20 °C).

Material	Thermal Conductivity	Standard Deviation
Neat PUF (0 wt%)	0.03031 W/m∙K	0.00003 W/m∙K
1 wt.% PUF-silica aerogel	0.03022 W/m∙K	0.00003 W/m∙K
3 wt.% PUF-silica aerogel	0.03109 W/m∙K	0.00010 W/m∙K
5 wt.% PUF-silica aerogel	0.03414 W/m∙K	0.00014 W/m∙K

## Data Availability

The data presented in this study are available on request from corresponding author.
